# Time-resolved nanospectroscopy of III–V semiconductor nanowires†

**DOI:** 10.1039/d5na00307e

**Published:** 2025-05-02

**Authors:** Andrei Luferau, Alexej Pashkin, Stephan Winnerl, Maximilian Obst, Susanne C. Kehr, Emmanouil Dimakis, Thales V. A. G. de Oliveira, Lukas M. Eng, Manfred Helm

**Affiliations:** a Institute of Ion Beam Physics and Materials Research, Helmholtz-Zentrum Dresden-Rossendorf Dresden 01328 Germany a.pashkin@hzdr.de; b Institut für Angewandte Physik, Technische Universität Dresden Dresden 01062 Germany; c Würzburg-Dresden Cluster of Excellence EXC 2147 (ct.qmat) Dresden 01062 Germany

## Abstract

We investigate ultrafast electron dynamics in individual GaAs/InGaAs core–shell nanowires using near-infrared pump–mid-infrared probe nanospectroscopy based on a scattering-type scanning near-field technique. Our results reveal a distinct blue shift in plasmon resonance frequency induced by photodoping. By extracting time-dependent electron densities and scattering rates, we gain insights into the effects of chemical doping and nanowire surface states on recombination dynamics and carrier mobility. Varying the pump power over two orders of magnitude reveals carrier recombination times in the range from a few ps at high power to 100 ps at low power, dominated by bimolecular recombination. Our findings highlight the potential of time-resolved nanoscopy for contactless probing of free carrier mobility and recombination dynamics on a local scale in individual semiconductor nanostructures or nanodevices.

## Abstract 2

We investigate ultrafast electron dynamics in GaAs/InGaAs core–shell nanowires. Our findings highlight the potential of time-resolved nanoscopy for contactless probing of carrier mobility and recombination dynamics in nanostructures.

## Graphical abstract

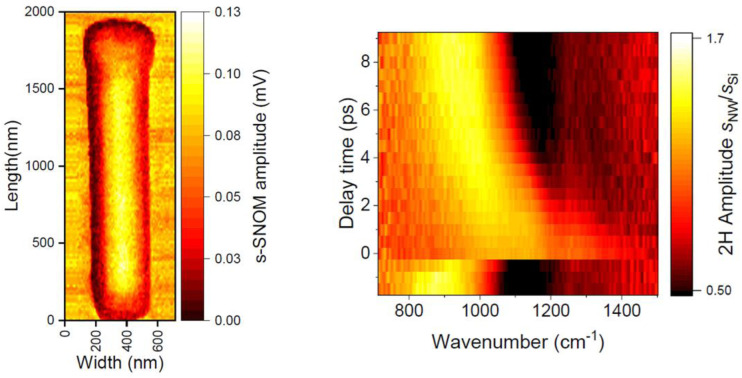

## Introduction

High-quality epitaxial nanowires (NWs) based on III–V semiconductors are of great interest as key components in future nano technology applications. These include sub-wavelength-sized lasers,^1^ photovoltaic cells with enhanced light absorption,^2^ tunnel field-effect transistors for energy-efficient electronic switching,^3^ and entangled photon-pair sources^4^ for quantum information technology. Additionally, single NWs may serve as highly sensitive elements in terahertz (THz) radiation detectors that operate at room temperature.^5,6^

To produce functional devices, it is essential to control the charge carrier concentration and mobility within NWs as part of precharacterization and quality control steps of production process. The THz time-domain spectroscopy (TDS) enables a contactless quantification of charge carrier density, mobility, and carrier lifetime in NW ensembles.^7,8^

Far-field THz conductivity spectra, however, are typically limited to large ensembles of NWs, as the spatial resolution is constrained by the THz radiation spot size. This diffraction-limited focus, determined by the THz wavelength *λ*_THz_, is several orders of magnitude larger than the dimensions of individual NWs. To overcome this limitation and enable localized, noninvasive characterization of single NWs, the scattering-type scanning near-field optical microscopy (s-SNOM) technique can be employed.^9^ Unlike far-field optical techniques, where the resolution is limited by diffraction, the near-field optical resolution is instead governed by the tip radius rather than the wavelength. In the case of THz radiation, s-SNOM has been shown to achieve spatial resolutions up to several thousand times smaller than *λ*_THz_.^10,11^

Several studies have demonstrated the feasibility of implementing NIR-pump/THz-probe techniques with near-field microscopy for the detailed examination of III–V NWs.^12–14^ These works, including our earlier study of THz-driven nonlinear carrier dynamics in highly-doped InGaAs NWs,^15^ have provided valuable insights into local carrier distributions, mobilities, and recombination dynamics within individual NWs. Due to the high surface sensitivity of s-SNOM, the reported results have often been attributed to surface-related processes, such as surface-defect-mediated recombination, the formation of surface carrier depletion layers, and diffusion effects. However, the overall understanding of these processes and their influence on the near-field response remains incomplete, highlighting the need for a more comprehensive in-depth investigation.

In this work, we address these gaps by conducting a series of NIR-pump MIR-probe nanospectroscopy experiments systematically varying key parameters such as NW chemical doping density and pump power. By examining the interplay of these factors, we are able to gain critical insights into how these factors influence both carrier scattering and recombination rates.

## Experimental

### Samples

Infrared nanospectroscopy studies in this work are performed on GaAs/InGaAs core–shell NWs grown by molecular beam epitaxy (MBE) on Si(111) substrates.^16,17^ These NWs feature a GaAs core with a diameter of 25 nm, surrounded by an 80 nm-thick In_0.44_Ga_0.56_As shell. The cross-sections of the NWs exhibit a hexagonal geometry, with the shell demonstrating a largely homogeneous composition both along and perpendicular to the NW axis.^17^ The core–shell structure facilitates controlled n-type doping with Si at varying concentrations.

Three types of NWs are studied here based on the shell's Si concentration: ‘undoped’ NWs with no intentional doping, ‘moderately-doped’ NWs with a nominal Si concentration of 2.8 × 10^18^ cm^−3^, and ‘highly-doped’ NWs with a nominal Si concentration of 8.2 × 10^18^ cm^−3^. Although the Si concentration for both ‘moderately-doped’ and ‘highly-doped’ NWs are generally considered heavy doping, this naming is used in this work to clearly differentiate doping levels in the analysis and discussion. For s-SNOM studies, these NWs are transferred onto a Si(100) substrate and dispersed randomly across it.

### Near-field nanospectroscopy setup

The near-field optical response of the NWs is investigated using an s-SNOM setup from Neaspec GmbH, equipped with a nano Fourier transform infrared (nano-FTIR) module. This module consists of an asymmetric Michelson interferometer and a broadband mid-infrared (MIR) difference-frequency generation (DFG) source (5–15 μm; *P*_avg_ ∼ 0.2 mW; 78 MHz; *t* < 120 fs), which serves as a probe.^18^ The second harmonic output of an erbium-doped fiber laser (775 nm; *P*_avg_ ≤ 13 mW; 78 MHz; *t* < 100 fs) is used as the near-infrared (NIR) pump.

**Fig. 1:**
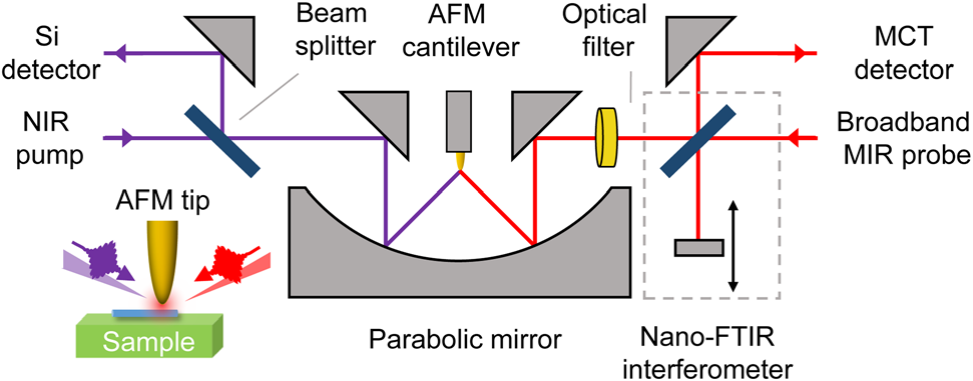
Schematic of the pump-probe s-SNOM setup integrated with a broadband nano-FTIR module. The MIR probe and external NIR pump beams are focused onto the AFM tip using a parabolic mirror.

Both the MIR and NIR beams are linearly p-polarized and are focused by an off-axis parabolic mirror (numerical aperture: 0.46, focal length: 11 mm) onto a metallized atomic force microscopy (AFM) tip, as shown schematically in Fig. 1. In particular, the parabolic mirror focuses the NIR pump beam (*P*_avg_ = 13 mW) with an initial beam diameter of ≈6 mm into a diffraction-limited spot of ≈2 μm, resulting in a fluence of *F* ≈ 7 mJ cm^−2^.

The parabolic mirror is also used to collect the radiation elastically scattered off the tip, causing the scattered NIR and MIR beams to propagate in multiple directions. Since the photoconductive mercury cadmium telluride (MCT) detector used for MIR detection is also sensitive to NIR radiation, a low-pass optical filter is placed in front of the interferometer to block the reflected NIR pump beam from entering the nano-FTIR optical path, thereby preventing detector saturation.

A Mylar polyester film is used as the beamsplitter for the pump radiation, offering high transmission for the NIR pump (*T*_NIR_ ≈ 90%) while reflecting a small fraction of the backscattered near-field signal toward a Si detector. Since the Si detector is insensitive to MIR radiation and the MIR probe power is relatively low, no additional filtering was necessary on this detection path.

Synchronization between the NIR pump and MIR probe is achieved by locking the probe source to the erbium-doped fiber laser oscillator. The time delay between pump and probe pulses can be adjusted *via* an optical delay line, enabling nano-FTIR spectra acquisition at each pump-probe delay.

To isolate the near-field signal from the far-field background, the AFM tip operates in tapping mode with an oscillation amplitude of approximately 100 nm. The signals from both the Si and MCT detectors are demodulated by a lock-in amplifier at the second harmonic of the AFM cantilever tapping frequency (*Ω*_0_ ≈ 250 kHz).^19^ After suppression of unwanted NIR components, the tip-scattered near-field MIR probe light interferes with the split MIR beam from the reference arm of the Michelson interferometer. The resulting signal is recorded as a function of reference mirror position,^20^ and the obtained interferograms are Fourier transformed to extract the spectra of the near-field scattering amplitude *s*(*ω*) and phase *φ*(*ω*).

## Results and discussion

### Nano-FTIR studies

**Fig. 2:**
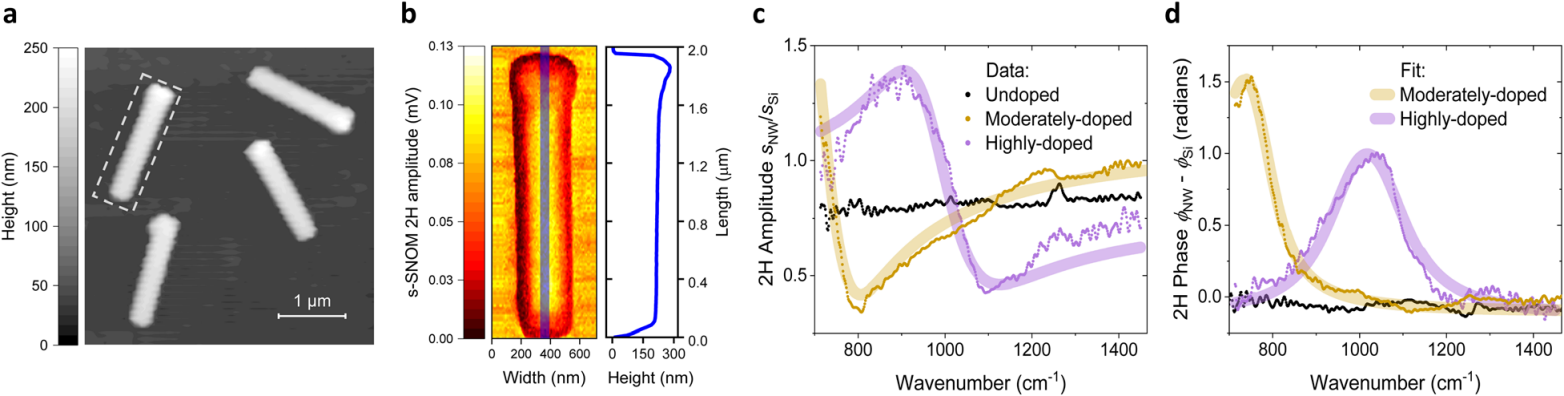
(a) AFM topography of dispersed NWs on Si(001) substrate. The white dashed rectangle highlights the NW selected for high-resolution mapping. (b) High-resolution s-SNOM amplitude map of the highly-doped NW on Si, acquired in ‘white light’ mode. The blue line marks the position of the longitudinal profile along the NW axis, shown alongside the map. (c) and (d) Near-field amplitude *s*(*ω*) and phase *ϕ*(*ω*) spectra of the highly-doped NW, measured at the center of the NW's top facet and normalized to the Si substrate response. The experimental data were fitted using a point-dipole model incorporating the frequency-dependent Drude–Lorentz permittivity, with fitting parameters for plasma frequency *ω*_pl_, scattering rate *γ*, and weight factor *c*.

Fig. 2(a) provides an AFM height overview of the dispersed highly-doped NWs on the Si(100) substrate, captured over a 5 × 5 μm^2^ scan area and a pixel resolution of 50 nm. This coarse mapping was performed using a quick scan in a serpent-like pattern, allowing efficient coverage of the larger surface with faster measurements to keep track of the dispersed NWs across the substrate. The white dashed rectangle highlights a single highly-doped NW chosen for high-resolution mapping, showing its orientation and position on the Si(001) substrate.

The result of the high-resolution s-SNOM scan of the highly-doped NW is shown in Fig. 2(b). Each pixel represents the amplitude of the nano-FTIR signal, which is demodulated at the second harmonic of the AFM cantilever tapping frequency. The measurements were conducted in ‘white light’ mode, in which broadband MIR light is detected with the reference arm of the nano-FTIR interferometer being blocked, enabling integral detection of the full spectral range of back-scattered light.

The blue line on the s-SNOM amplitude map indicates the position of the longitudinal profile along the NW axis, shown together with the s-SNOM map in Fig. 2(b). The profile exhibits a prominent height peak corresponding to a remnant droplet at the top of the NW, a characteristic feature of nanowires grown by the vapor–liquid–solid (VLS) mechanism during MBE growth.^16^

The homogeneous bright area in the amplitude map originates from the NW's top facet, where the signal is strongest. This uniform amplitude distribution is attributed to the plasmonic response of the doped carriers in the InGaAs shell, creating a distinct contrast between the NW and the surrounding Si substrate. Darker regions in the amplitude map appear where the facets of the NW are inclined or shadowed, reducing the detected signal in these areas. To prevent shadowing and other geometry-related artifacts spectrally resolved measurements are performed with the tip positioned at the center or the top facet (see Fig. 2(b)).

Fig. 2(c) and (d) present the results of spectrally resolved nano-FTIR studies on NWs with different doping levels, acquired in the standard nano-FTIR mode at a demodulation order of *n* = 2. Both the amplitude spectra, *s*(*ω*) = *s*_NW_(*ω*)/*s*_Si_(*ω*), and the phase spectra, *φ*(*ω*) = *φ*_NW_(*ω*) – *φ*_Si_(*ω*), are normalized to the response of the Si substrate, assuming its spectrally flat response over the photon energy range of the MIR probe.^21^

The highly and moderately doped NWs exhibit a pronounced resonance behavior in both the amplitude *s*(*ω*) and phase *φ*(*ω*) spectra, characteristic of the plasmonic response in doped semiconductors. As the carrier density decreases, a distinct shift toward lower frequencies is observed, accompanied by an increase in *φ*(*ω*). For undoped NWs, the plasmonic resonance shifts beyond the probing range of the MIR source, resulting in a spectrally flat response. A minor feature appears around 1250 cm^−1^, likely originating from the tip antenna resonance, which is present in all spectra and cannot be fully eliminated through reference normalization. This feature is consistently present in all spectra and cannot be fully eliminated through reference normalization.

### Near-field modeling

To quantify the near-field plasmonic response of the NWs, we employ a point–dipole model^22^ that relates the tip-scattered field *E*_sca_ to the incident field *E*_in_ through a complex-valued scattering coefficient:1
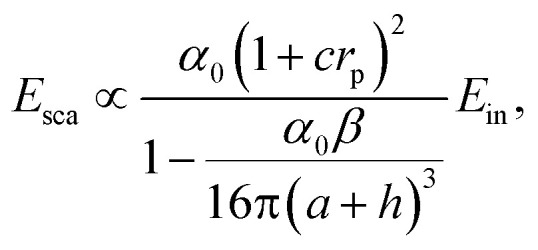
where *α*_0_ = 4πa^3^ represents the polarizability of the tip (with radius *a*) induced by the incident field *E*_in_. Here, *h* represents the tip-apex-sample separation, and *β* = (*ε* − 1)/(*ε* + 1) denotes the quasi-electrostatic local near-field reflection coefficient of the sample. The parameter *r*_p_ is the Fresnel reflection coefficient for p-polarized light, while *c* is a weighting factor that quantifies the contribution of the surface-reflected beam relative to the direct tip illumination.^23^ Since the focused MIR beam area is estimated to be several times larger than the NW, most of the surface reflection is expected to originate from the Si substrate. Therefore, we approximate *r*_p_ using the Fresnel coefficient of Si.

Higher harmonic demodulation is incorporated by considering the sinusoidal oscillation of the tip *h*(*t*) and applying Fourier transformation to *E*_sca_[*h*(*t*)]. This results in *E*_*n*_ ∝ *s*_*n*_*e*^*i*^*^ϕn^*, where *s*_*n*_ and *ϕ*_*n*_ represent the amplitude and phase of the scattered electric field at demodulation order *n* = 2, respectively.

The permittivity dispersion *ε*(*ω*) is approximated using the Drude model:2
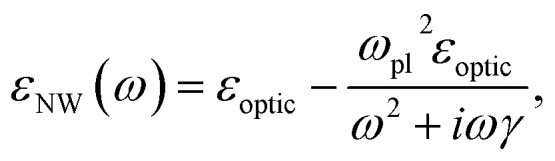
where *ε*_optic_ is the high-frequency permittivity. The second term describes the response of free carriers, with the plasma frequency *ω*_pl_ and the electron scattering rate *γ* given by:3
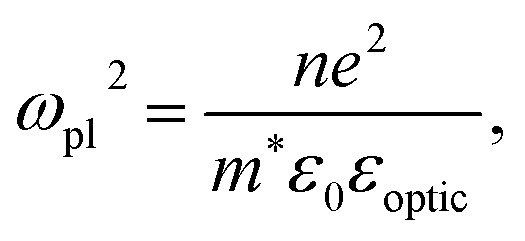
4
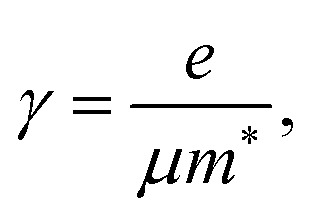
with *e* the elementary charge, *ε*_0_ the vacuum permittivity, *n* the electron concentration, and m* and *μ* the effective mass and mobility of the charge carriers, respectively.

In our modeling approach, we incorporate the interferometric detection scheme by assuming that the reference beam is identical to the field incident on the tip *E*_in_. Under this assumption, the intensity measured by the MCT detector is expressed as *I* ∝ |*E*_sca_||*E*_in_|*e*^*i*(*ϕ*_sca_ − *ϕ*_in_)^.

Consequently, normalizing the near-field signal from the NW to the Si reference removes dependence on the incident field *E*_in_ while also compensating for variations in optical alignment, detector spectral sensitivity, and other instrumental factors that might otherwise influence *E*_sca_. As a result, the normalised near-field plasmonic response is fully described by just three physical parameters: the plasma frequency *ω*_pl_, scattering rate *γ*_pl_, and weight factor *c*.

The model was applied to simultaneously fit the experimental near-field amplitude and phase spectra. The results, shown in Fig. 2(c) and (d) as curves plotted above the data, accurately reproduce the spectral features observed in the experiment. The shift in the plasma resonance is quantified by the difference between the plasma frequencies of highly-doped (*ω*_pl_ = 1025 cm^−1^) and moderately-doped (*ω*_pl_ = 740 cm^−1^) NWs. The observed increase in *φ*(*ω*) is primarily attributed to a reduction in the scattering rate, with the moderately-doped NW exhibiting *γ* = 85 cm^−1^, compared to *γ* = 170 cm^−1^ for the highly-doped NW. At the same time, the difference in weight factors (*c* = 0.85 for moderately-doped *vs. c* = 0.50 for highly-doped) is attributed to variations in alignment and geometrical configuration between the two NWs during measurements.

Given that the InGaAs shell is significantly thicker than the GaAs core and has a smaller band gap compared to GaAs, we neglect core–shell effects and carrier transfer to the core. Instead, we approximate the NW response as the plasmonic response of the heavily n-doped InGaAs shell alone. The nonparabolicity of the InGaAs *Γ*-valley conduction band (CB) is accounted for by considering the energy-dependent effective mass and the nonparabolic carrier distribution, leading to a density-dependent variation of the average effective mass m*(*n*).^24^ The estimation relies on two key parameters: the effective mass at the *Γ*-point (*m*_*Γ*_ = 0.043*m*_e_),^25^ and the nonparabolicity parameter, defined as *α* = 1/*E*_g_,^26^ where *E*_g_ = 0.835 eV corresponds to the band gap of In_0.44_Ga_0.56_As.^25^

By applying m*(*n*) in eqn (3) and using the experimentally obtained values of *ω*_pl_, we determine a Fermi energy of *ε*_F_ = 260 meV at 300 K for the highly doped NW, corresponding to a total electron density of *n* = 8.1 × 10^18^ cm^−3^. This value is in close agreement with the nominal Si dopant concentration of 8.2 × 10^18^ cm^−3^, indicating efficient incorporation of Si dopants into the InGaAs lattice, each contributing by one free electron.^16^

Similarly, for the moderately doped NW, we obtain a Fermi energy of *ε*_F_ = 170 meV at 300 K, corresponding to a total electron density of *n* = 3.8 × 10^18^ cm^−3^, which is also in reasonable agreement with the implemented Si doping concentration.

The average effective mass m* for the electron densities *n* = 8.1 × 10^18^ cm^−3^ and *n* = 3.8 × 10^18^ cm^−3^ is determined to be 0.06*m*_e_ and 0.055*m*_e_, for highly- and moderately-doped NWs respectively. Determining m* further allows us to convert the fitted scattering rates *γ* = 170 cm^−1^ and *γ* = 85 cm^−1^ into the corresponding mobilities. Using eqn (4), we obtain carrier mobilities of *μ* = 900 cm^2^ V^−1^ s^−1^ for the highly doped NW and *μ* = 3400 cm^2^ V^−1^ s^−1^ for the moderately doped NW.

### NIR-pump MIR-probe nanospectroscopy

**Fig. 3:**
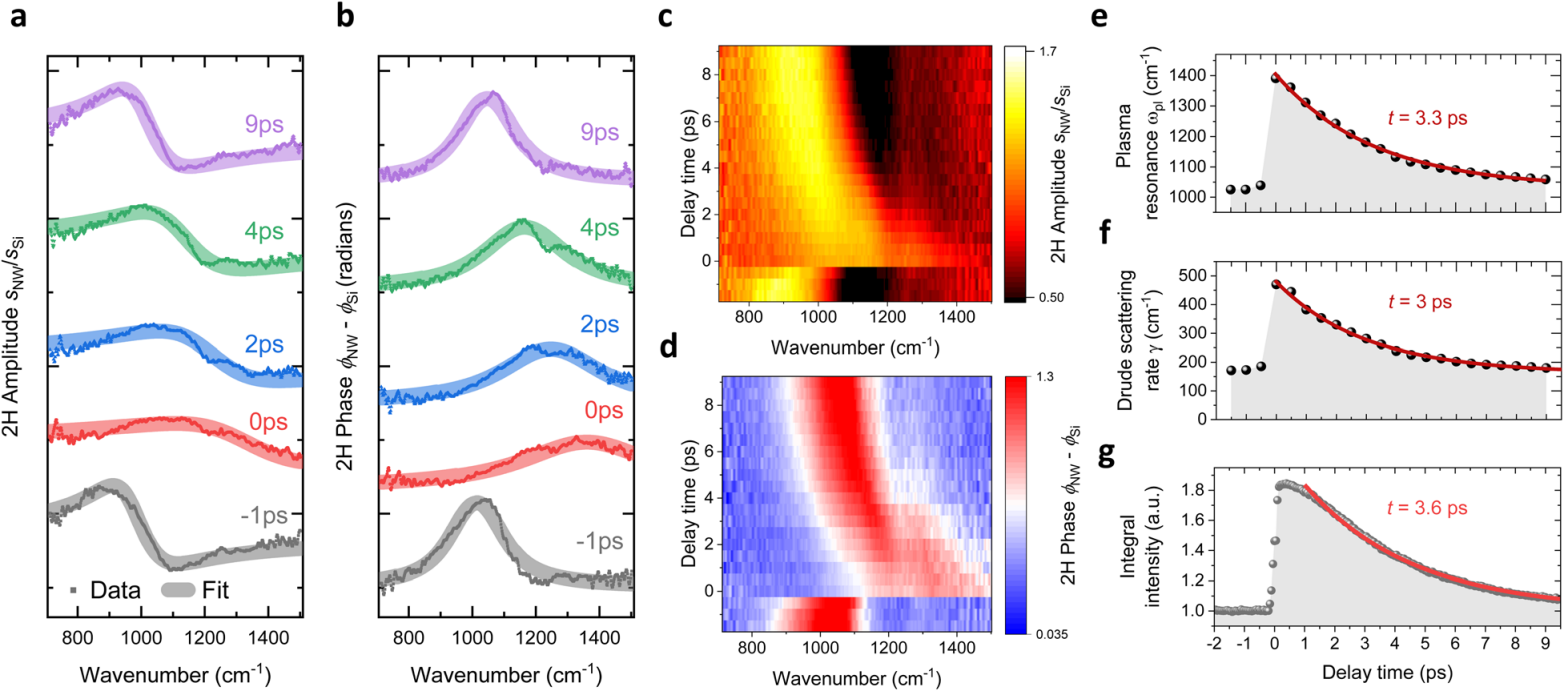
(a) and (b) the near-field amplitude *s*(*ω*) and the phase *ϕ*(*ω*) spectra of the highly-doped NW, measured at the different delay times between nano-FTIR probe and NIR-pump (*P*_avg_ = 13 mW) and normalized to the response of the Si substrate. The plotted spectra correspond to a few representative time delays, and are fitted using a three-parameter point-dipole model based on the frequency-dependent Drude–Lorentz permittivity. (c) and (d) Color maps illustrating the evolution of *s*(*ω*) and *ϕ*(*ω*) upon NIR photoexcitation over the entire range of pump-probe delay times, with each line representing normalized near-field spectra at different delays. (e) and (f) Fitted values of the plasma resonance frequency *ω*_pl_ and the scattering rate *γ*_pl_ as functions of pump-probe delay time, showing decay times of *t* = 3.3 ps and *t* = 3 ps, respectively. (g) Pump-induced change in the spectrally integrated scattered intensity as a function of pump-probe delay time for the highly-doped NW, normalized to the unpumped baseline.

The NIR-pump MIR-probe nanospectroscopy is employed to track the temporal evolution of the plasmonic response of carriers under non-equilibrium conditions. The results for the highly-doped NW are presented in Fig. 3. Upon NIR photoexcitation (*P*_avg_ = 13 mW), the plasma resonance shifts toward higher frequencies, accompanied by broadening of both the near-field amplitude *s*(*ω*) and phase *ϕ*(*ω*) spectra. This effect is evident in the spectra corresponding to a few representative time delays, as shown in Fig. 3(a) and (b). As the pump-probe delay increases, the plasma resonance gradually returns to its original position, and the spectral shapes recover their equilibrium state. It is worth noting that due to the high photoexcitation intensity and potentially poor thermal coupling between the NW and the substrate, laser heating may raise the average NW temperature by tens (possibly hundreds) of Kelvins above room temperature.

The complete temporal evolution of *s*(*ω*) and *ϕ*(*ω*) over the entire range of pump-probe delays is illustrated in the color maps in Fig. 3(c) and (d), where each line represents near-field spectra at a specific delay time, normalized to the response of the Si substrate. To quantify significant transient changes observed in *s*(*ω*) and *ϕ*(*ω*) the three-parameter point-dipole model implemented, similar to the approach used for the unpumped case. However, the factor *c*, which represents the relative weight of the surface-reflected beam to the direct tip illumination, was assumed to remain constant regardless of photoexcitation and was constrained to its unpumped value.

The fitted near-field spectral response is shown as curves overlaid the data in Fig. 3(a) and (b), accurately capturing the spectral modifications observed for each time delay. The extracted plasma resonance frequency *ω*_pl_ and scattering rate *γ* as functions of pump-probe delay time are presented in Fig. 3(e) and (f). Both parameters are increased upon NIR excitation, and then decay monoexponentially, with fitted time constants of *t* = 3.3 ps for *ω*_pl_ and *t* = 3.0 ps for *γ*.

Additionally, the results of the spectrally integrated pump-probe measurements are shown in Fig. 3(g). In this method, the nano-FTIR reference arm is blocked (see Fig. 1) and the signal is demodulated at the second harmonic of the cantilever frequency similar to the spectrally-resolved measurement. This method detects the total intensity of the broadband scattered light at each pump-probe time delay. The resulting pump-probe curve is normalized to the baseline at negative delay times for better representation, and reveals a significant change in the spectrally integrated signal upon photoexcitation. A monoexponential fit of its decay yields a time constant of *t* = 3.6 ps, which is comparable to the decay time of *ω*_pl_ and *γ*.

Although this method lacks spectral resolution, it still provides valuable insights into carrier dynamics. The resemblance of its decay dynamics with that of *ω*_pl_ demonstrates that it can be used for a rough evaluation of decay time. Additionally, the spectrally integrated acquisition facilitates faster data acquisition as compared to interferometric measurements, making it a beneficial pre-characterization tool for determining characteristic time scales of carrier dynamics within the NWs. Furthermore, it enables the collection of larger datasets while maintaining consistent experimental conditions, such as optical alignment and AFM tip condition, across all measurements. In following, we employ this method for power-dependent measurements.

### Temporal evolution of electron densities

The photoinduced changes in plasma resonance are considered to be dominated by electrons, since their effective mass (*m*_*Γ*_ = 0.043*m*_e_) in In_0.44_Ga_0.56_As is much smaller than the heavy-hole mass of *m*_hh_ = 0.44*m*_e_,^25^ resulting in less contributions to the plasmonic response. We also neglect optical transitions from the light-hole band since the density of states compared to the heavy-hole band is small.

**Fig. 4:**
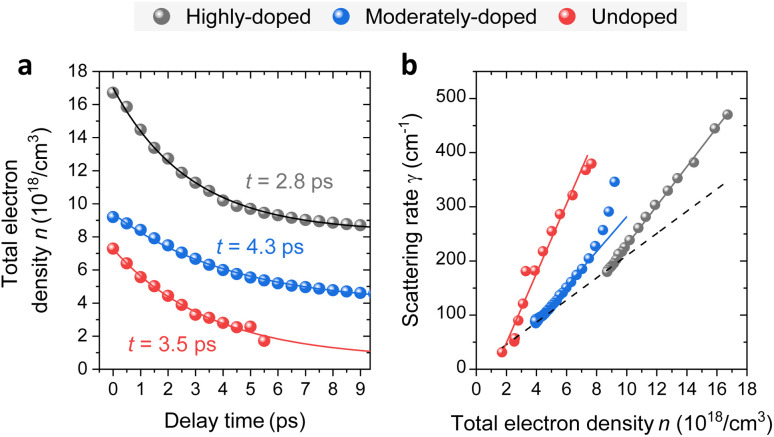
(a) Temporal evolution of the total electron density *n* for the highly-doped, moderately-doped, and undoped NWs, showing an exponential decay with time constants of *t* = 2.8 ps, *t* = 4.3 ps, and *t* = 3.5 ps, respectively. (b) Scattering rate *γ* as a function of total electron density *n* for the highly-doped, moderately-doped, and undoped NWs. The solid lines approximate the linear density dependence of the total scattering rate upon photoexcitation. The dashed line connects the points corresponding to the minimal electron density for each NW type, highlighting chemical doping impact on the scattering rate.

Under this assumptions we derive from experimental values of *ω*_pl_ [shown in Fig. 3(e)] the electron distributions and corresponding electron densities for each pump-probe time delay, accounting for the non-parabolicity effect in the same way as was done under unpumped conditions. The results are shown in Fig. 4(a), where *n* is plotted as a function of pump-probe time delay following photoexcitation for the high-doped NW along with results for the moderately-doped and undoped NWs. The data points corresponding to other NWs are acquired in the same manner as for highly-doped NW, and detailed outcomes of the related pump–probe nanospectroscopy experiments are available in the ESI.†

The total electron density *n* is composed of the intrinsic electron doping density *n*_d_ and the photogenerated carrier density Δ*n*. By using *n*_d_ values obtained from nano-FTIR measurements in the absence of photoexcitation, we determine Δ*n* for the highly and moderately doped NWs. In the undoped NW case, where no intrinsic doping is present, *n* is directly equal to Δ*n*, which remains below 10^19^ cm^−3^, as in doped NWs. While this provides a general reference, a direct comparison of Δ*n* between different NWs is not particularly meaningful, as it is influenced by various factors, including absorption, geometrical configuration, and alignment variations, which are beyond the scope of this discussion.

Following photoexcitation, the carrier population in the doped NWs relaxes toward the doping density *n*_d_, with monoexponential fits yielding time constants of *t* = 2.8 ps for the highly-doped NW and *t* = 4.3 ps for the moderately-doped NW. In the undoped NW, the photogenerated carriers decay to their intrinsic background level—which is several orders of magnitude lower—with a time constant of *t* = 3.5 ps.

### Density dependence of scattering rate

The evolution of the scattering rate *γ*(*n*) is analyzed in correlation with the total electron density *n* in the photoexcited NWs, yielding the carrier density dependence of the scattering rate *γ*(*n*) in the photoexcited NWs, as shown in Fig. 4(b).

To investigate variations in *γ*(*n*) among different NW types, we consider contributions from both bulk-like and surface scattering mechanisms, which become particularly significant in low-dimensional structures such as NWs due to their high surface-to-volume ratio.^27–29^ According to Matthiessen's rule, the total scattering rate can be expressed as:

5 *γ* = *γ*_i_ + *γ*_eh_ + *γ*_ph_ + *γ*_s_,

where *γ*_i_ represents impurity scattering due to doping, *γ*_eh_ accounts for electron–hole interactions, *γ*_ph_ describes electron-phonon scattering, and *γ*_s_ denotes surface scattering processes.

For undoped NWs, electron scattering is primarily dominated by surface interactions and Fröhlich-mediated longitudinal optical (LO) phonon scattering, the latter being the dominant mechanism in polar semiconductors.^30^ In doped NWs, impurity scattering *γ*_i_ significantly contributes due to ionized impurities.^31^ The effect of doping is approximated by a linear trend, connecting *γ*(*n*) data points at minimal carrier densities for the doped and undoped NWs, as indicated by the dashed line in Fig. 4(b).

Upon photoexcitation, *γ*(*n*) increases quasi-linearly, consistent with previous studies on both bulk GaAs^32,33^ and GaAs NWs.^28,34^ Moreover, the slopes in Fig. 4(b) are steeper than the doping-only trend (dashed line), indicating additional scattering contributions, mainly from electron–hole interactions *γ*_eh_. Photoexcited holes with a larger effective mass, serve as nearly elastic scattering centers for electrons, leading to an additional increase in *γ*(*n*).^35^

Notably, the moderately-doped NW exhibits a deviation from the linear *γ*(*n*) dependence at higher carrier densities. A similar behavior was observed for one of the highly-doped NWs measured in the additional dataset (see ESI†), suggesting that this phenomenon is not limited to a specific doping level. Although all highly-doped NWs are expected to share similar bulk properties, the observed differences in *γ*(*n*) may indicate variations in surface conditions. Given the high surface sensitivity of s-SNOM, these deviations are likely linked to changes in surface scattering contributions. Such differences may have originated from the mechanical transfer process, which could modify the energy or density of surface states. These alterations, in turn, may influence the local surface electric field, thereby enhancing the surface scattering.

Although the observations reveal a significant influence of surface scattering, a conclusive attribution to specific surface states would require complementary studies, such as surface passivation experiments or Kelvin probe microscopy, which are beyond the scope of the present work. The primary objective of this study was to investigate carrier dynamics.

### Power-dependent dynamics of photoexcited carriers

**Fig. 5:**
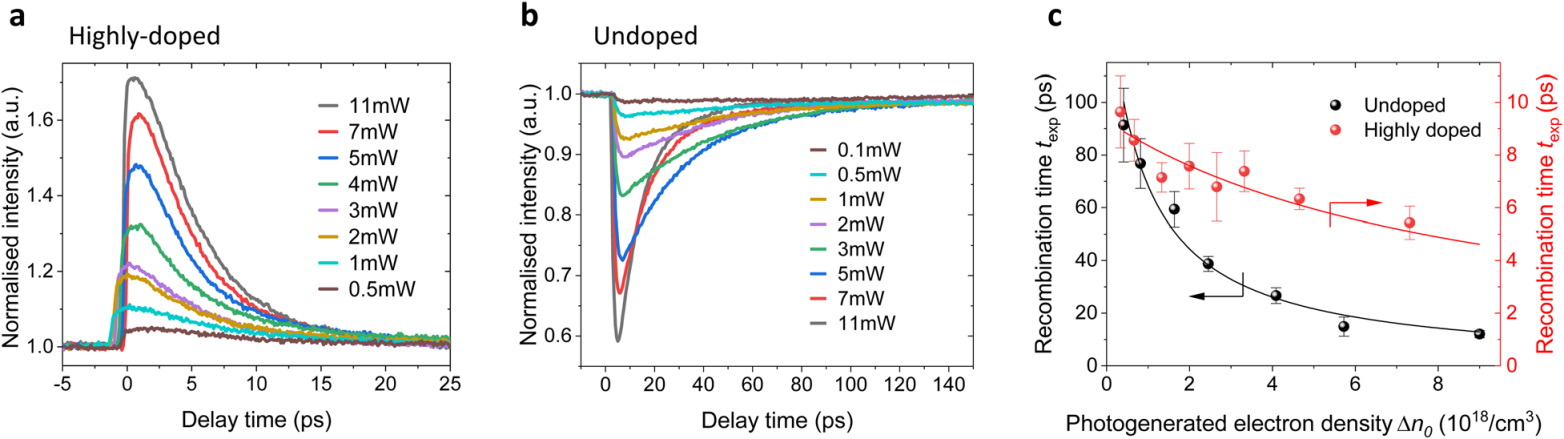
Pump-induced changes in the scattered intensity as a function of pump-probe delay time and pump power for highly-doped (a) and undoped (b) NWs. All pump-probe traces are normalized to the unpumped baseline. (c) 1/*e* decay times extracted from monoexponential fits of the pump-probe traces for each NW type as a function of photogenerated electron density Δ*n*.

To gain deeper insights into carrier recombination dynamics and the influence of power-dependent effects, we conducted a series of spectrally integrated pump-probe scans on the undoped and highly-doped NWs, maintaining consistent experimental conditions, including optical alignment and AFM tip condition. In these measurements, the reference nano-FTIR arm was blocked, and the pump power was varied using a set of neutral density filters, enabling systematic variation of the pump power while ensuring uniformity across experiments. Fig. 5 presents the pump-induced changes in scattered intensity as a function of pump-probe delay time and pump power for the highly doped (a) and undoped (b) NWs. All pump-probe traces are normalized to the baseline at negative delay times.

Upon photoexcitation, spectrally integrated pump-probe traces exhibit an initial change of opposite sign. This reflects the qualitative difference in the impact of photoexcitation in both cases: in the undoped NWs the photoexcitation shifts the plasmon closer to the probe frequency, but it still remains below the middle of the spectral range resulting in a lowering of the integrated signal. The difference between the amplitude spectra for the undoped and moderately doped NWs in Fig. 2(c) can be used as an analogy for the cases of unpumped and pumped NW, respectively. In the highly-doped NW the situation is opposite. The plasmon is located within the probing spectral range in the equilibrium state and the photoexcitation shifts it to higher frequencies beyond the probe range. This results in an increase in the total signal.

In the moderately-doped NW, the behavior is more complex since the plasmon, located at the low edge of the probing range in equilibrium (Fig. 2(c)), shifts into the middle of the range upon photoexcitation and returns during relaxation process (see Fig. S2 in ESI†). As a result, the integrated pump-probe signal shows an initial increase followed by a drop to negative values (Fig. S2(f) in ESI†), making it an unreliable indicator of recombination dynamics in this case. Therefore, we focus on undoped and highly-doped NWs in the subsequent analysis of recombination dynamics.

To quantify the relationship between the spectrally integrated signal and the actual carrier dynamics, we introduce a scaling factor *η*, that relates *t*_exp_ obtained from spectrally integrated traces (for example, in Fig. 3(g)) to the actual decay of photogenerated electron density (Fig. 4(a)). This is particularly relevant, because in the following power-dependent analysis, we will rely only on spectrally integrated decays. Based on our measurements, we find *η* ≈ 1.3 for the highly doped NW and *η* ≈ 2 for the undoped NW. The difference in the *η* factors stems from the photoexcitation impact on the plasmon position in the undoped and highly-doped NWs with respect to the probing spectral range as outlined above. Besides the sign of the spectrally integrated pump-probe signal it also influences its relation to the plasmon frequency *ω*_pl_ leading to the difference in the scaling of the relaxation time.

The recombination dynamics of photogenerated electrons in a semiconductor can be described by the differential equation:^36^6
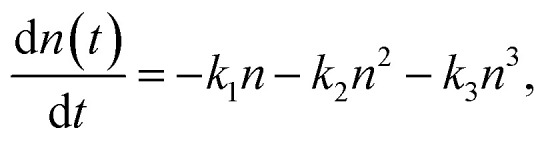
where *n*(*t*) represents the electron density as a function of time *t* following photoexcitation. The decay constant *k*_1_ characterizes the monomolecular recombination *via* defects and surface states, *k*_2_ is the radiative (bimolecular) rate constant, while *k*_3_ is the constant describing Auger processes.^37^

In bulk GaAs, Auger recombination becomes significant for carrier densities exceeding *n* > 2.5 × 10^19^ cm^−3^.^38^ Since this is beyond the carrier densities considered in our study here, we neglect Auger recombination and, instead, account for chemical *n*-doping of the NWs with a doping electron density *n*_d_. Under these conditions, eqn (6) simplifies to:^31^7
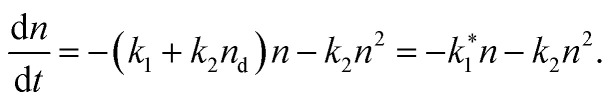


All experimental pump-probe traces are well-described by a monoexponential decay, with positive traces fitted for the highly doped NW and negative traces for the undoped NW. To ensure consistency across all measurements, the fits were performed from the maximum signal until it reaches half of its initial value. The reason for this choice will be discussed later. The extracted decay times *t*_exp_ at each pump power were then correlated with the corresponding photogenerated electron density Δ*n*_0_.

To determine Δ*n*_0_, nano-FTIR spectra were measured at the temporal overlap of the pump and probe pulses at *P*_avg_ = 13 mW. By fitting the resulting plasma resonance shift, we extracted carrier densities of Δ*n*_0_ = 10.6 × 10^18^ cm^−3^ for the undoped NW and Δ*n*_0_ = 8.7 × 10^18^ cm^−3^ for the highly doped NW. Assuming a linear absorption approximation, these values were extrapolated across the full range of applied pump powers.

Since the recombination times at low pump powers differ by nearly one order of magnitude between doped and undoped NWs, we visualize *t*_exp_ as a function of Δ*n*_0_ in Fig. 5(c), using two separate *Y*-axes: the left axis corresponds to the undoped NW, while the right axis represents the highly-doped NW.

Bimolecular recombination is often neglected in studies on III–V NWs.^8,31,34^ However, in our case, this assumption was insufficient to describe the observed power-dependent changes in recombination dynamics. To account for the gradual decrease in *t*_exp_ with increasing pump power, we implemented the analytical solution of eqn (7), given by:8
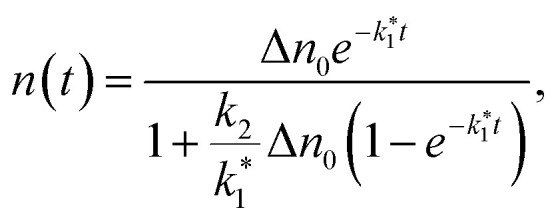
where Δ*n*_0_ represents the density of photogenerated electrons immediately after excitation.

For short times (*t* → 0), we use the approximation 
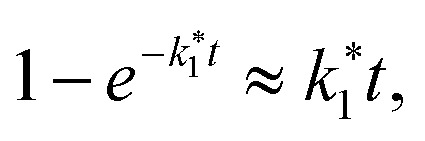
 simplifying the denominator to 1 + *k*_2_Δ*n*_0_*t*. Further applying the first-order approximation for *k*_2_Δ*n*_0_*t* ≪ 1, we express the denominator as ek^_2_^Δn^_0_^t. This yields an exponential approximation of eqn (8) for short times as follows:9
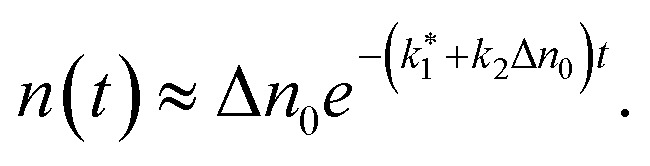


From this, we derive the density-dependent carrier recombination lifetime for spectrally-integrated studies to:10
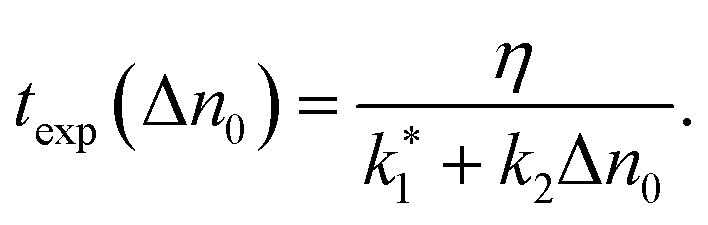


The above-mentioned scaling factor *η* is introduced here because eqn (9) is derived for the decay of the photogenerated electron density, whereas the experimentally measured decay corresponds to the spectrally integrated signal. Since our monoexponential fits of the pump-probe traces were performed from the maximum signal down to half of its initial value, the use of eqn (10) is justified, as it describes the initial stage of the carrier recombination dynamics. We simultaneously fitted the experimental density dependencies of *t*_exp_ for both NW types, using a shared *k*_2_ parameter. The fits yield 
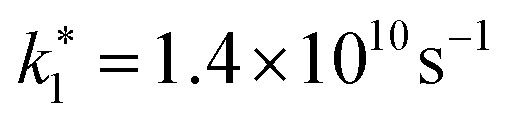
 for the undoped NW, 
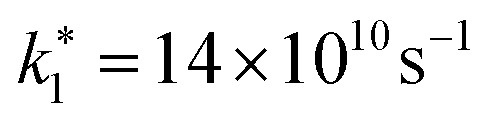
 for doped NWs, and *k*_2_ = 1.5 × 10^−8^ cm^3^ s^−1^ is equal for both NWs. The corresponding fit curves are overlaid the experimental data in Fig. 5(c), showing good agreement and confirming the bimolecular nature of the observed power-dependent decrease in *t*_exp_.

Using the carrier concentration *n*_d_ = 8.1 × 10^18^ cm^−3^ for the highly doped NW, extracted from the nano-FTIR plasma resonance fit under equilibrium conditions, and estimating the intrinsic carrier concentration in the undoped NW as *n* ≈ 10^16^ cm^−3^, we can derive the actual values of *k*_1_ from the relation 
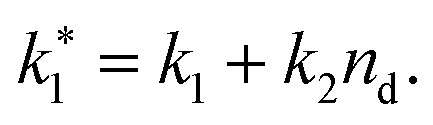
 For the undoped NW, due to the low intrinsic carrier density, 
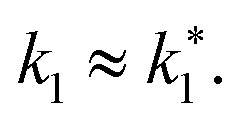
 For the doped NW, we find *k*_1_ = 1.8 × 10^10^ s^−1^. The higher value of *k*_1_ in the doped NW is consistent with an increased density of impurities and trap states introduced by doping. However, since the change in *k*_1_ is relatively modest compared to the variation in 
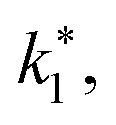
 we main difference in *t*_exp_ between doped and undoped MWs s is attributed to the increased free electron concentration resulting from doping.

At low pump powers, *t*_exp_ for the undoped NW approaches values previously obtained from far-field NIR-pump THz-probe studies of similar In_0.44_Ga_0.56_As NWs, where carrier lifetimes of approximately 100 ps were observed.^39^ In those experiments, the applied fluence of *F* ≈ 15 μJ cm^−2^ was two to three orders of magnitude lower than in our focused-beam experiment. This lower fluence corresponded to an estimated photogenerated carrier density of Δ*n*_0_ ≈ 2 × 10^17^ cm^−3^, resulting in an approximately proportional decrease in carrier density. This trend suggests that the ps-time scale dynamics observed in our study—as well as in previously reported NIR-pump MIR-probe nanospectroscopy studies on III–V semiconductors^12,40^—originates from the high fluences required to excite plasma resonances into the MIR spectral range.

While the bimolecular model successfully captures the overall recombination behavior, the exact role of surface states in this process remains unclear. Additional studies specifically targeting surface properties would be beneficial to further clarify the contribution of surface-mediated recombination. Notably, a nearly twofold difference in recombination rates between nominally identical highly-doped NWs, measured under identical conditions, may point to a significant role of surface states (see Fig. S3†). Given the high surface-to-volume ratio of NWs, variations in surface conditions likely influence carrier trapping dynamics and may underlie the observed pump-power-dependent recombination behavior. In this context, the bimolecular recombination model may serve as an effective approximation if surface states dynamically fill and their occupation depends on both electrons and holes.

## Conclusions

In this work, we have demonstrated the use of NIR-pump MIR-probe nanospectroscopy to investigate the plasmonic response and carrier dynamics in GaAs/InGaAs core–shell NWs with varying doping levels. Through nano-FTIR measurements, we systematically examined the near-field optical response, revealing distinct shifts in plasma resonance driven by both doping variations and photoexcitation-induced carrier dynamics. By employing a three-parameter point-dipole model, we extracted the carrier concentration and scattering rate, further estimating their temporal evolution through recombination dynamics, providing a quantitative insight into doping-dependent electronic properties.

The power-dependent pump-probe measurements revealed a systematic decrease in carrier recombination lifetime with increasing excitation power, which we attribute to an enhancement in bimolecular recombination both *via* the introduction of new carriers through photoexcitation and chemical doping. Furthermore, the observed behavior may also be explained by a non-saturable surface-mediated electron–hole recombination process, highlighting the intricate balance between bulk and surface recombination pathways.

At low pump powers, the extracted lifetimes approach values reported in far-field NIR-pump THz-probe studies on similar In_0.44_Ga_0.56_As NWs, where carrier relaxation times of approximately 100 ps were observed. At higher powers, the observed ps-time scale carrier recombination dynamics in our study – as well as in previous NIR-pump MIR-probe nanospectroscopy studies – likely originate from the significantly higher excitation fluences required to induce a detectable plasmonic response in the MIR spectral range.

These findings contribute to the broader understanding of charge carrier dynamics in III–V semiconductor nanostructures, providing a foundation for optimizing NW-based optoelectronic and quantum devices. Furthermore, our study underscores the potential of near-field nanospectroscopy as a high-resolution, non-invasive technique for characterizing the electronic properties of nanoscale materials.

## Data availability

Data for this article, including raw data as well as processed Origin files and Python scripts for modeling and fitting, are available at the Rossendorf Data Repository (RODARE) at https://doi.org/10.14278/rodare.3654. In addition, the data supporting this article have been included as part of the ESI.†

## Author contributions

Andrei Luferau: investigation, data curation, formal analysis, writing – original draft. Alexej Pashkin, Stephan Winnerl, Susanne C. Kehr, Manfred Helm: scientific discussion, supervision. Maximilian Obst, Thales V. A. G. De Oliveira: investigation. Emmanouil Dimakis: resources (Sample Provision), scientific discussion. Lukas M. Eng: supervision. All authors contributed to writing – review editing and have given approval to the final version of the manuscript.

## Conflicts of interest

The authors declare that there are no competing interests.

## Supplementary Material

NA-OLF-D5NA00307E-s001
