# Supplementary material for: Time-resolved nanospectroscopy of III–V semiconductor nanowires
Source: Nanoscale Adv. 2025 May 2;7(12):3692–700. doi: 10.1039/d5na00307e (PMC12060794; doi:10.1039/d5na00307e)
Supplement: NA-007-D5NA00307E-s001 [file NA-007-D5NA00307E-s001.pdf]

Supplementary Information (SI) for Nanoscale Advances.
This journal is © The Royal Society of Chemistry 2025

Electronic Supplementary Information

for "Time-resolved nanospectroscopy of III-V semiconductor nanowires"

Andrei Luferau,[ab] Alexej Pashkin,[a] Stephan Winnerl,[a] Maximilian Obst,[bc] Susanne C. Kehr,[bc] Emmanouil Dimakis,[a] Thales V. A. G. de Oliveira,[a] Lukas M. Eng,[bc] and Manfred Helm[ab]*

[a] *Institute of Ion Beam Physics and Materials Research, Helmholtz-Zentrum Dresden-Rossendorf, Dresden 01328, Germany*
[b] *Institut für Angewandte Physik, Technische Universität Dresden, Dresden 01062, Germany*
[c] *Würzburg-Dresden Cluster of Excellence, EXC 2147 (ct.qmat), Dresden 01062, Germany*

*Corresponding author: a.pashkin@hzdr.de

S1. Near-field pump-probe studies on undoped and moderately-doped nanowires

Supplementary Fig. S1(a, b) shows color maps illustrating the temporal evolution of the near-field amplitude $s(\omega)$ and phase $\phi(\omega)$ spectra of the undoped GaAs/InGaAs core-shell NW upon NIR photoexcitation. Each line corresponds to the near-field spectrum at a specific pump-probe delay time, normalized to the response of the Si substrate. As the undoped NW exhibits no plasmonic resonance within the probing MIR range under equilibrium conditions, we analyze its transient response following excitation, fitting only those spectra where the plasmonic resonance clearly appears within the detectable spectral range using the three-parameter point-dipole model (see main text for details).

The extracted temporal evolution of the plasma resonance frequency ω_{pl} and scattering rate γ are shown in Fig. S1(c, d). Both parameters exhibit a monoexponential decay, with extracted time constants of $t = (6 \pm 2)$ ps for ω_{pl} and $t = (11 \pm 5)$ ps for γ. However, the uncertainty in the fits is relatively high, primarily due to the limited time window in which the plasmonic resonance remains within the MIR probing range.

In parallel, Supplementary Fig. S1(e) presents results from a spectrally integrated pump-probe measurement, acquired by blocking the nano-FTIR reference arm. The resulting time trace exhibits an initial short-lived positive peak, attributed to the excitation of ω_{pl} near the center frequency of the broadband probe ($\omega_0 \approx 1120$ cm^{-1}). The following negative tail is well described by a monoexponential decay, with a time constant that closely matches the decay of ω_{pl}, providing a useful complementary view of the transient response.

Supplementary Fig. S2(a, b) presents similar color maps for the moderately doped NW. Again, each spectrum is normalized to the Si substrate and fitted using the point-dipole model. The extracted dynamics of ω_{pl} and γ, shown in Fig. S2(c, d), reveal a clear increase upon photoexcitation followed by monoexponential relaxation back to the equilibrium state, with fitted time constants of $t = 4.9$ ps for ω_{pl} and $t = 2.8$ ps for γ.

The corresponding spectrally integrated pump-probe trace, shown in Fig. S2(e), exhibits only a minor negative tail following the initial positive peak. This negative feature is significantly weaker than that observed in the undoped NW. This behavior can be attributed to the fact that ω_{pl} in the moderately doped NW remains within the MIR probing range throughout the entire measurement window, thereby preventing complete spectral suppression of the resonance. Additionally, the decay of the integrated signal differs significantly from that of ω_{pl}, so it does not reliably reflect the recombination dynamics in this case.

This effect becomes even more pronounced in the power-dependent measurements, as shown in the inset of Fig. S2(f), where a reduction in pump fluence leads to further suppression of the negative tail. Due to the poor agreement between the spectrally integrated and spectrally resolved dynamics in the moderately doped NW, we exclude the integrated measurements from the analysis presented in the main text.

S2. Variations in scattering dynamics among highly-doped NWs

We additionally investigated a set of highly-doped NWs using NIR-pump MIR-probe nanospectroscopy. The results for two representative nanowires, labeled NW2 and NW3, are presented in Fig. S3. Here, the total electron concentration n is derived by converting the fitted plasma frequency ω_{pl}, while the scattering rate γ_{pl} is directly obtained from the fitting process using the three-parameter point-dipole model. The total electron density n as a function of pump-probe delay time for NW2 and NW3 shows monoexponential fits yielding time constants of $t = 1.8$ ps and $t = 3.3$ ps, respectively.

The carrier density dependence of the scattering rate $\gamma(n)$ for each NW are derived by correlating the temporal evolutions of n

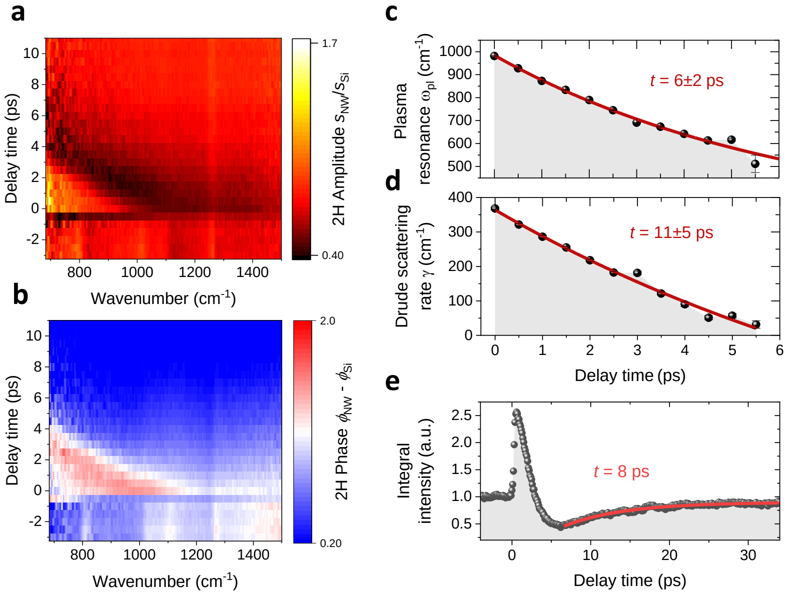

Figure S1 Results for the undoped NW. (a,b) Color maps illustrating the evolution of the near-field amplitude $s(\omega)$ and the phase $\phi(\omega)$ spectra upon NIR photoexcitation, measured at the different delay times between nano-FTIR probe and NIR-pump ($P_{\rm avg}$ =13 mW) and normalized to the response of the Si. Only spectra exhibiting a clear plasmonic resonance within the MIR range were fitted using a three-parameter point-dipole model based on the frequency-dependent Drude-Lorentz permittivity. Each spectra are fitted using a three-parameter point-dipole model based on the frequency-dependent Drude-Lorentz permittivity. (c, d) Fitted values of the plasma resonance frequency ω_{pl} and the scaterring rate γ_{pl} as functions of pump-probe delay time. (e) Pump-induced change in the spectrally integrated scattered intensity as a function of pump-probe delay time for the undoped NW, normalized to the unpumped baseline.

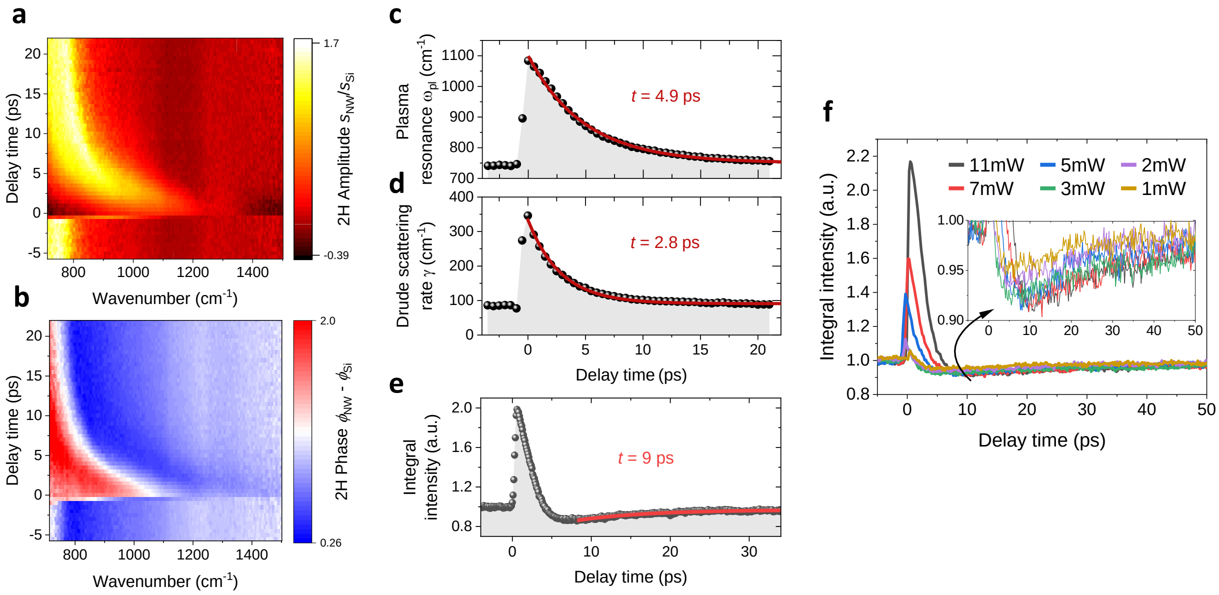

Figure S2 Results for the moderately-doped NW. (a,b) Color maps illustrating the evolution of the near-field amplitude $s(\omega)$ and the phase $\phi(\omega)$ spectra upon NIR photoexcitation, measured at the different delay times between nano-FTIR probe and NIR-pump ($P_{\rm avg}$ =13 mW) and normalized to the response of the Si. Each spectra are fitted using a three-parameter point-dipole model based on the frequency-dependent Drude-Lorentz permittivity. (c, d) Fitted values of the plasma resonance frequency ω_{pl} and the scaterring rate γ_{pl} as functions of pump-probe delay time. (e) Pump-induced change in the spectrally integrated scattered intensity as a function of pump-probe delay time, normalized to the unpumped baseline. (f) Power-dependent changes of spectrally-integrated pump-probe trace for moderately-doped NW. The inset in (f) provides a closer view of the negative signal tail that follows the initial short positive peak.

and γ. Fig. S3(c) compares the results for NW2 and NW3 with those of NW1 (from Fig. 4 of the main text). While NW1 and NW2 demonstrate consistent linear $\gamma(n)$ dependencies, NW3 shows a noticeable change in linear behavior at higher photoexcited carrier densities, marked by a steeper $\gamma(n)$ slope, as illustrated by the dashed line in Fig. S3(c).

The observed differences in scattering behavior for NW3 compared to the other highly-doped NWs are likely attributed to variations in surface conditions, as these NWs are expected to have similar bulk properties. These variations may have arisen from the mechanical transfer process, potentially altering the energy or density of surface states. Such changes could modify the surface electric field increasing electron scattering on surface.

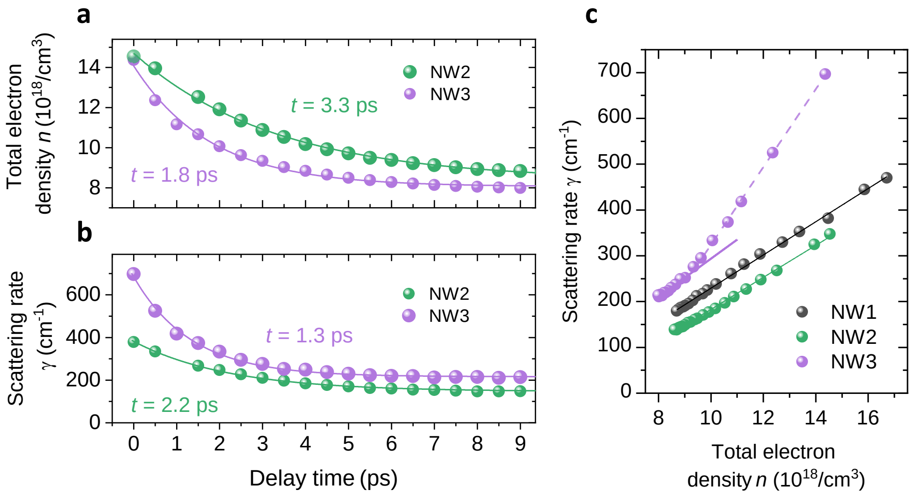

Figure S3 (a) Results for the highly-doped NWs. Total electron density n as a function of pump-probe delay time for NW2 and NW3, with monoexponential fits yielding time constants of $t = 1.8$ ps and $t = 3.3$ ps, respectively. (b) Electron scattering rate γ as a function of delay time for NW2 and NW3, showing fitted decay times of $t = 1.3$ ps and $t = 2.2$ ps, respectively. (c) Scattering rate γ as a function of total electron density n for NW1, NW2, NW3. While NW1 and NW2 demonstrate consistent linear $\gamma(n)$ dependencies, NW3 shows a noticeable change in linear behavior at higher photoexcited carrier densities, marked by a steeper $\gamma(n)$ slope.
